# Effectiveness of English Online Learning Based on Deep Learning

**DOI:** 10.1155/2022/1310194

**Published:** 2022-04-13

**Authors:** Jie Xu, Yang Liu, Jinzhong Liu, Zuguang Qu

**Affiliations:** ^1^Department of College English Teaching and Researching, Qiqihar University, Qiqihar 161006, China; ^2^Foreign Language Department, Qiqihar Medical University, Qiqihar 161006, China

## Abstract

With the popularization of the Internet lifestyle and the innovation of learning methods, more and more online learning systems have emerged, allowing users to study in the system anytime and anywhere. While providing convenience to users, online learning systems also bring troubles to users, who cannot quickly find the resources they are interested in from the huge amount of learning resources. In this paper, we apply deep learning to an English online learning platform and analyze learners and learning contents by clustering algorithm and association rules. Based on this, a content organization system is developed using genetic algorithms, which is applied to the case of this paper to provide learners with personalized learning content. With the hope that the system can be extended to other online learning platforms in the future, three data mining techniques were selected to solve the problems found in the English online learning platform, and we designed how these techniques should be applied to the online learning platform. The first technique is the cluster mining technique, which is used to analyze learners' profiles, classify learners in different categories, provide them with personalized learning content, and organize group learning. The second technique is association rules, which is used to analyze the correlation between learning contents. For the adaptive student-teacher knowledge migration strategy, the teacher model can guide the student model to track online and migrate the task-specific knowledge to the online tracking student model through the network parameters. Finally, a case study is selected and the above design is applied to this case study, and the results are analyzed in detail. The data mining technology is applied to the English online learning platform, and an innovative English learning content organization system is developed. It is hoped that the results of this study will have some practical value for promotion and provide an idea for the construction of the online learning platform, and it is also expected that the idea can improve the quality of online learning to a certain extent. Specifically, the online student model is adaptively updated by the teacher model parameters and the online student model parameters together.

## 1. Introduction

Personalized learning is an important way to realize students' personalized development, an important goal for school construction, and an important direction for education development. With the in-depth application and rapid development of information technology in the field of education and the rapid promotion of the “Internet + Education” strategy, online learning resources have also been developed at a high speed, and we can get the learning resources we are interested in anytime and anywhere through the Internet terminal [1]. However, while the increasing number of online learning resources brings us opportunities, it also brings us challenges in personalized learning [2]. The huge amount of learning resources can easily cause us to be lost in resources, and we cannot find the most suitable learning resources among many learning resources quickly [3]. Deep learning technology, speech recognition technology, face recognition technology, and other intelligent technologies have been widely used in other fields, bringing numerous conveniences to our production life and getting good evaluations [4]. Therefore, how to use intelligent technology to realize personalized recommendations of learning resources has become a hot and difficult research point in the field of education. Personalized learning can promote the development of “Internet + education” and is also of great significance in the actual process of education and teaching. Personalized learning is widely accepted and loved by learners [5]. Personalized learning is the basic need for efficient learning, which can effectively combine learners' individual characteristics and provide targeted guidance to learners; personalized learning is also an important direction of teaching reform, which can fully reflect the learner-centered thinking and provide opportunities for students' personalized development [6–9].

Personalized learning is an inevitable trend in the development and progress of education, which can improve the shortcomings of existing education and provide the possibility of personalized development of education [10]. With the rapid development of Internet technology, online education is welcomed and loved by the majority of learners because of its convenience without the limitation of time and space and is also favored and valued by the capital market as well as major institutions and schools, and online education platforms have sprung up like a spring [11]. At the same time, in order to meet the diversified learning needs of different learners, online education platforms have been increasing the construction of online learning resources, so that we can obtain the colorful learning resources we need for free anytime and anywhere through networked devices, which brings us numerous convenience in learning. The Internet has provided us with many rich and diverse learning resources that can be shared and reused, and Internet technology has facilitated our learning with resources, which is the benefit and opportunity given to us in the Internet era [12–15]. Opportunities and challenges coexist. With the rapid growth and accumulation of Internet resources, the massive and diverse learning resources easily bring about problems such as information disorientation and lead to excessive redundant information, making it difficult for learners to distinguish the authenticity of learning resources [16]. It takes a lot of time and effort for learners to find the learning resources they need and like the most from the vast amount of learning resources related to their learning interests, and it also requires learners to have certain information recognition and screening ability, which is a challenge for learners and an even bigger challenge for beginners. Therefore, we have to meet the challenge and rise to the challenge to solve the problem of disorientation brought by the richness of learning resources in the Internet era and to realize personalized learning of learning resources [17].

Since the application of deep learning technology in the field of education started late, although there are more studies on the personalized recommendation of learning resources, there are fewer studies on the personalized recommendation of learning resources combined with deep learning technology. This study summarizes the characteristics of personalized recommendation of learning resources, constructs a deep neural network-based personalized recommendation model of learning resources, and designs the corresponding algorithm by fully analyzing the advantages and shortcomings of the existing related research, thoroughly studying the related theories and technologies, which can provide a theoretical reference for the personalized recommendation of learning resources supported by deep learning technology and describe how to realize personalized recommendation. This study gives full play to the advantages of deep learning technology, constructs a personalized recommendation model of learning resources supported by deep learning technology, and applies the recommendation model to a personalized recommendation platform of learning resources based on a deep neural network to collect users' real evaluations after trying the platform. This can provide an effective reference for the functional module setting, data recording, and analysis of online learning platforms and promote online learning platforms to realize personalized recommendation of resources and improve user experience and satisfaction, which has a reference value for the construction and development of online learning platforms.

## 2. Deep Learning-Assisted Personalized Learning of English

### 2.1. English Personalized Learning

Personalized learning recognizes the individual characteristics and differences of each student and promotes the personalized development of each student by implementing individualized learning plans, learning paths, and learning strategies so that each student can experience and personalize learning [18]. Personalized learning requires us to recognize that each student is an individual with different learning styles, learning rhythms, learning paths, and learning aspirations, to support multiple learning choices and opportunities, and to meet the individual learning needs of each student. Traditional personalized learning, due to limitations in technology and resources, mainly consists of teachers formulating different instructional plans according to the different characteristics of different learners and learners realizing personalized learning by completing personalized tasks formulated by teachers, but this does not really meet the personalized needs of learners [19]. The return of artificial intelligence technology in the era of artificial intelligence, with machine learning and deep learning as the key support, has reshaped and rebuilt personalized learning, and “Internet + education” enables learners to obtain the learning content they need regardless of time and space, and “artificial intelligence + education “AI + Education” further provides personalized services for learners.

Personalized learning in the age of intelligence can strengthen learner-centered thinking through technological means, emphasize learners' personalized learning environment, learning time, and learning methods, respect learners' different cognitive abilities, interest preferences, learning speed, and other personalized characteristics, and enable students to obtain personalized learning experience and learning effectiveness, so as to better promote learners' lifelong personalized development. Personalized learning has the main features of personalization, autonomy, and flexibility. It starts with each learner, considers each learner's different learning style, cognitive level, and learning interests, and provides personalized learning content and learning methods for each learner through intelligent technology methods. This study is a personalized recommendation of learning resources, which includes personalized learning content for learners. Therefore, this study will build a personalized recommendation model for learning resources based on deep neural networks for learners with different cognitive abilities, different learning interests, and different learning styles.

The action *a* *=* *(∆a, ∆b)* consists of an action involving a target movement, as follows:

(1) $$\begin{array}{c} \Delta a=\alpha q^{t},\\ \Delta b=\alpha p^{t}, \end{array}$$

where *α* is set 0.3 according to ADNet experience.

The state st is represented by the information inside the tracking box and the dynamic action generated by the action dynamic vector together:

(2) $$\begin{array}{c} b=\left(a_{1},a_{2},q^{t},p^{t}\right), \end{array}$$

denotes the target position coordinates and the length and width of the tracking frame, respectively. Specifically, a preprocessing function,

(3) $$\begin{array}{c} s=\phi \left(q^{t},f\right), \end{array}$$

is designed to crop the image block in the tracking frame *q*^*t*^ in the specified frame *f*.

The state transfer function is used to describe the horizontal and vertical changes of the target. The tracking algorithm moves the target tracking frame to a new position according to the predicted action. The tracking frame *b*_*t*_ is moved to the new position *b*_*t* *+* 1_ according to the action *a*_*t* *+* 1_, which can be expressed as follows:

(4) $$\begin{array}{c} q=a_{t}+a_{t+1},\\ p_{t}=b_{t}+b_{t+1}, \end{array}$$

unlike other existing deep reinforcement learning-based tracking algorithms that use a simple strategy to explore the action space, thus enhancing the exploration capability of the tracking algorithm.

The reward function *r*(*s*, *a*) is used to evaluate the transfer by taking the action *a* and transferring the state *s* to the state *s*′ afterward. It is obtained from the overlap ratio (IoU) between the predicted tracking frame *b*_*t*_ and the true target frame *G*, as shown in the equation.

**Figure 1:**
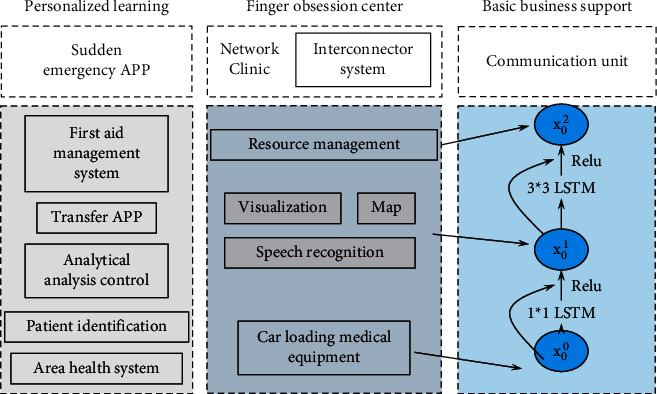
Personalized learning algorithm pretraining process.

In the proposed target tracking algorithm framework, the mainstream target tracking framework is followed. As shown in Figure 1, a random variance descent gradient based on nonconvex optimization is designed as a backpropagation method for alleviating the local optimal solution problem in target tracking. In addition, an action reward loss function is also designed for training the tracking algorithm, which implements Intersection Over Union (IoU) between the predicted tracking frame and the real tracking frame.

(5) $$\begin{array}{c} \min\;f\left(x\right)=f\left(x-1\right)+g\left(a\right), \end{array}$$

where *f* (*x*) denotes the smoothing function and *g*(*x*) denotes the regularizer. Currently, most deep reinforcement learning-based target tracking algorithms use SGD to solve the backpropagation problem as shown in the equation. where *θ* denotes the model parameters, B denotes the minibatch, *g* denotes the regularizer, *η* denotes the learning rate, and *t* denotes the number of iterations:

(6) $$\begin{array}{c} \eta_{t+1}=\eta_{t}-\frac{\theta_{t}}{\sum_{i=1}\Delta f_{i}\left(t\right)}. \end{array}$$

However, SGD methods usually require more time for algorithm convergence because of the large variance generated by ∇*f*_*i*_(*θ*_*t*_) during random sampling. A sequence-based hyperparametric optimization method is used to improve the algorithm identification. The existing estimates are averaged and the approximation error variance is reduced. Three averaging strategies are proposed for reducing the estimation bias. A combination of cross-entropy and double-delay depth-deterministic strategy gradients is used to enhance the algorithm's robustness. To further improve the accuracy and computational efficiency of the tracking algorithm, a nonconvex optimized random variance descent gradient is introduced instead of the SGD method for backpropagation, which is expressed as

(7) $$\begin{array}{c} \beta_{i+1}=\beta_{t}-\left(\frac{\eta_{t}}{\sum_{i=1}\Delta f_{i}\left(t\right)}+f_{i}\left(q\right)\right). \end{array}$$

Assuming that the current training epoch is *s*, a copy of the model parameters will be generated from the previous training epoch. Thus, it can reduce the variance of the model parameter update, as shown in Table 1.

Due to the action exploration space limitation, deep reinforcement learning-based target tracking algorithms usually suffer from the problem of falling into local optimal solutions. When the algorithm falls into the local optimal solution, the limited action exploration space will restrict the algorithm to jump out of the local optimal solution, resulting in tracking failure. However, existing deep reinforcement learning-based target tracking algorithms use a simple action space search strategy to try to solve this problem but still fall into a locally optimal solution. To solve this problem, an adaptive exploration method is proposed, which uses spatiotemporal information for optimizing the action space search, enabling the algorithm to jump out of the local optimal solution and thus find a better solution. This method extends the action space using spatiotemporal information and improves the performance of the tracking algorithm by balancing exploitation and exploration.

### 2.2. Online Target Tracking and Deep Learning Strategies

Before the emergence of these target tracking video datasets, researchers could only test tracking algorithms by their own filmed samples; no unified dataset was available, resulting in the inability to measure and compare algorithms with each other [20]. The emergence of OTB and VOT datasets, for example, provides a unified test data platform and defines many metrics for measuring algorithm performance, providing a complete process for testing and comparing the performance of relevant tracking algorithms, further accelerating the performance improvement of target tracking algorithms. The designed target tracking algorithms are also trained and tested on OTB, VOT, and GOT-10k datasets and compared with other State-Of-The-Art (SOTA) tracking methods. The details of the relevant datasets are shown in Table 2, and the relevant datasets are presented separately next.

Target tracking algorithms are evaluated for performance by measuring the distance difference between the predicted position and the true target position. Robust tracking algorithms are able to maintain the distance between the predicted position and the true target position within a small range:

(8) $$\begin{array}{c} \chi =\left\|x_{t}-a\left(t-1\right)\right\|, \end{array}$$

where *G*_*t*_ denotes the coordinates of the center point position of the real target in frame *t*-th, *O*_*t*_ denotes the coordinates of the center point position of the target tracking frame predicted by the tracking algorithm in the frame, and the calculation result represents the center error of frame in the video sequence. Center errors can reflect the accuracy of the location prediction of the target tracking algorithm in a specific frame of the tracking sequence. If all center errors in a video sequence are used for mapping, the center error map of the tracking algorithm can be retrieved for this video sequence.

The accuracy of the target is evaluated by the central error and the central error threshold TC is used to assess whether the target tracking algorithm has been successfully followed. In the definition of tracking success based on the center error for the center error, *f*_*t*_ tracking is considered successful. Therefore, the correct response rate is the percentage of the number of frames successfully followed relative to the total number of frames, and the correct response rate is the percentage of frames with a central error of less than Tc for the total number of frames, where Tc is generally interpreted as follows: pixel point. The specific calculation formula is shown as follows:

(9) $$\begin{array}{c} P_{R}=\frac{\partial p-q^{t}}{N}. \end{array}$$

Regional reviews can only evaluate the tracking performance for a specific frame, not the overall performance of a video sequence. The expected mean overlap (EAO) is based on overlapping the areas of each frame of the video sequence, and the overall robustness of the tracking algorithm for the entire video sequence is evaluated by calculating the average overlap of all areas of the video sequence. The specific formulae are

(10) $$\begin{array}{c} \theta =\frac{\sum_{t=1}\partial p-q^{t}}{N}. \end{array}$$

**Figure 2:**
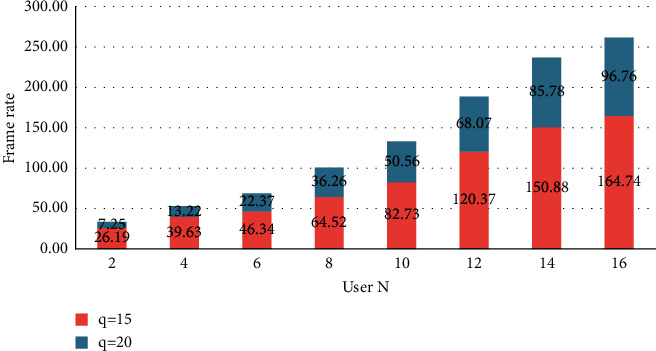
Frame rate threshold change curve.

The success rate of the target is defined by the overlap of the range. If the range overlaps in the definition of the overlap success, the track success is considered to be successful. Therefore, the success rate is the percentage of successful frameworks in relation to the total number of frames. The success rate is the percentage of frames where the range overlaps with the total number of frames greater than *N*. The specific formulae are

(11) $$\begin{array}{c} s=\frac{\sum_{t=1}q^{t}}{N}, \end{array}$$

where *q* specifies the number of frames that fulfill condition *N* and *N* specifies the total number of frames in the video sequence. So, the higher the success rate, the better the tracking performance of the tracking algorithm. If the size of the mean error threshold changes, the success rate also depends on the threshold value. Therefore, the success rate curve can be obtained by displaying the threshold value and the corresponding correct response rate. If you increase the threshold gradually, the success rate curve becomes a monotonous increasing curve, as the number of frames fulfilling the condition increases gradually, as shown in Figure 2. At the same time, the performance of the proposed method in high-speed movements is somewhat lower than in some mainstream trackers.

### 2.3. Research on the Effectiveness of Online Learning

**Figure 3:**
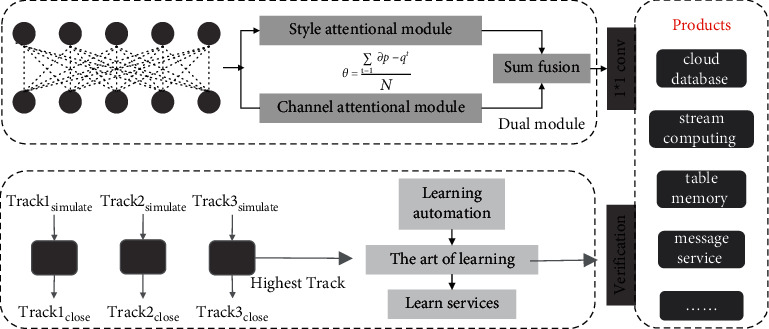
Trace algorithm to verify validity.

Existing tracking algorithms based on deep reinforcement learning use action space noise to improve the exploration ability of models in complex scenes [21]. However, action space noise is generated completely randomly, which is stochastic and nonreproducible. Excessive randomness will lead to dramatic fluctuations of the tracking algorithm in complex scenes and thus lose the target. In addition, the action space noise is an approximately linear function, which is difficult to fit complex functions and limits the tracking performance of the tracking algorithm in complex scenes. To solve this problem, parametric space noise is introduced to the target tracking field for the first time. Inserting noise in the parameter space can enhance the exploration ability of the algorithm, thus further improving the robustness of the tracking algorithm in complex scenes, as shown in Figure 3. While ensuring the robustness of the tracking algorithm in complex scenes, it can also generate richer action behaviors to improve the exploration capability of the tracking algorithm.

In the model, the model training level and resource recommendation level are closely interlinked, the model training level is the basis of the resource recommendation level, the resource recommendation level is the application of the model training level, and the main purpose is to improve the accuracy and timeliness of learning resource recommendation after learning through big data. The resource filtering module reduces the number of learning resources in the resource recommendation module and reduces the complexity of the resource recommendation module; the resource recommendation module provides personalized learning resources for the resource display module, which can improve the efficiency of the learning resource recommendation process and improve the accuracy, diversity, initiative, and timeliness of learning resource recommendation. Compared with traditional recommendation models and methods, the model uses deep learning technology, adds resource filtering methods and resource display methods, makes certain optimization in terms of recommendation efficiency, display effect, and learning initiative, and reduces the complexity of the model, in order to improve the sense of efficiency and satisfaction of personalized recommendation of learning resources.

**Figure 4:**
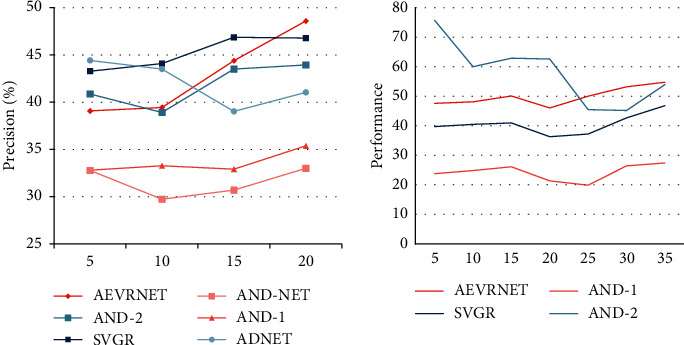
Experimental results for different tracking challenges.

These attributes of OTB dataset benchmarks have also been analyzed to further analyze the robustness of the proposed tracking algorithm over the challenges.  Figure 4 shows the results of all tracking algorithms for the eight main video attributes of ope in the OTB100 dataset. The proposed AEVRNet method is characterized by light changes, low resolution, and background arrangement. In comparison to ADNet, AEVRNet exceeds ADNet in scale variation and in-plan rotation with accuracy and success rates of 2.6%, respectively. 4.1%. Since the proposed method uses regression rather than classification, it is more sensitive to changes in various aspects of the target state, e.g., the width and height of the target. Compared to ECO and MDNet, the proposed AEVRNet uses adaptive search to improve the larger action space and has the ability to jump out of locally optimal solutions. This result is related to the feature extraction overhead of deep neural networks. This is because the built-up network uses fewer layers, improves tracking speed, and limits the detectability of deep features.

## 3. Results and Analysis

### 3.1. Effectiveness of Tracking Learning

**Figure 5:**
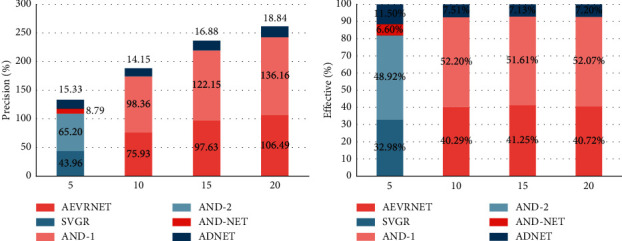
Effectiveness of tracking learning.

In order to further analyze the contribution of each component of the algorithm, various combinations of the proposed methods at OTB100 and OTB2013 were evaluated. ADNet was used as a benchmarking algorithm. SVRG shows that the proposed method uses only a combination of nonconvex optimization variances. There are descent cases for offline training and online tracking. SVRG action shows that the proposed method uses a combination of nonconvex optimized random dispersion degrees and adaptive search for target [22]. AEVRNet shows that the proposed method uses a combination of all nonconvex optimized random dispersion degrees, adaptive search, and regression-based training.  Figure 5 shows the performance of all these combinations. You can see that everyone is gradually improving the performance of the proposed method. For adaptive searching, the combination of temporal and spatial relationships to solve visual tracking problems based on deep reinforcement learning during online tracking improves the search and successfully emerges from local optimal solutions. The results show that the two data sets improve by 2.9% and 5.0%, respectively. The regression loss function reduces the loss of information and improves the robustness of the proposed method. For the problems found in the English online learning platform, three data mining techniques are selected to solve the problems found, and how these techniques should be applied in the online learning platform is designed.

**Figure 6:**
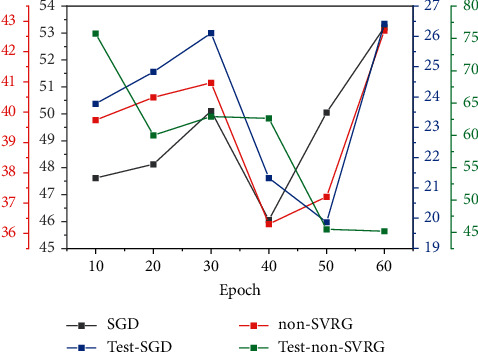
Training loss of the proposed method and testing error of pretraining.

In order to further analyze the contribution of each component of the algorithm, various combinations of the proposed methods at OTB100 and OTB2013 were evaluated. ADNet was used as a benchmarking algorithm [23]. SVRG shows that the proposed method only uses a combination of nonconvex optimized random dispersion degrees for offline training and online tracking. SVRG action shows that the proposed method uses both nonconvex optimized random dispersion degrees as well as adaptive combinations. There is reconnaissance for target pursuit. AEVRNet shows that the proposed method uses a combination of all nonconvex optimized random dispersion degrees, adaptive search, and regression-based training.  Figure 6 shows the performance of all these combinations. You can see that everyone is gradually improving the performance of the proposed method. The results show that the two data sets improve by 2.9% and 5.0%, respectively. The regression loss function reduces the loss of information and improves the robustness of the proposed method.

**Figure 7:**
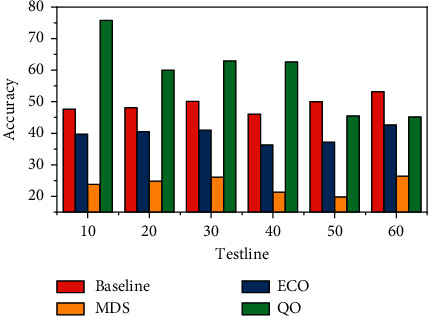
Results of the benchmark method with nonconvex optimization on OTB100.

We analyze the impact of the proposed nonconventional randomly dispersed optimization gradient to show specific reasons why nonconventional optimization gradients can improve the performance of the target audience algorithm. The training of the 120 s keeps the same settings as with ADNet. Nonconvex optimized random dispersion gradients can converge quickly with losses of 0017 or 0027 in the training and test data sets. The nonconvex optimized random dispersion gradient initializes the parameters of the next epoch using the best solution for the current training epoch. The training speed of the model can be accelerated compared to SGD using random parameters as initial parameters. Nonconvex optimization random dispersion gradients in both datasets can improve the accuracy of benchmark algorithms by 0.8% and 0.6%. In order to further analyze the effectiveness of the proposed nonconvex optimized random dispersion gradient, it is applied to deep learning-based trackers ECO and MDNet. As shown in Figure 7, the nonconvex optimized random dispersion gradient offers improvements of 0.6% and 0.5%, respectively, in ECO and MDNet. This is mainly due to the ability of the nonconvex optimized random dispersion gradient to converge faster with less loss during training and to improve the accuracy of the proposed method. Extensive experiments on six generic target tracking datasets show that the proposed method outperforms other deep reinforcement learning-based tracking methods.

## 4. Analysis of Experimental Results

**Figure 8:**
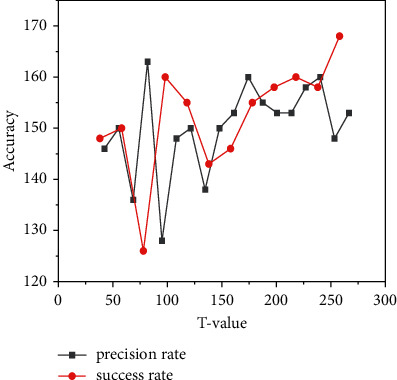
Results for different *T* of the dataset.

The effect of hyperparameter *T* in adaptive action exploration was also analyzed. Improvements of 2.3% and 3.1% were obtained on both datasets. The analysis of the results in Figure 8 shows that adaptive exploration can effectively mitigate the occurrence of target loss in occlusion and ambiguity cases. To further analyze the effect of *µ* on action selection, three action selection methods were designed on OTB100. Compared with the greedy strategy, the current mean can obtain 1.1% and 0.8% improvement in accuracy and success rate, respectively. When ${\hat{\mu }}_{i}$ is combined with the standard deviation, it can improve 0.6% and 0.5% in precision and success rate, respectively. For adaptive searching, the combination of temporal and spatial relationships to solve visual tracking problems based on deep reinforcement learning during online tracking improves the search and successfully emerges from local optimal solutions.

**Figure 9:**
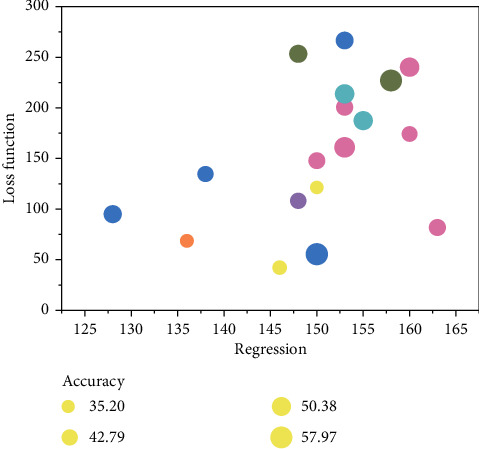
Recommendation learning accuracy.

The impact of the regression-based loss function on training is analyzed. It achieves a 0.6% and 2.0% improvement on the two datasets, respectively, because the regression-based loss function makes the algorithm more sensitive to changes in the target state, such as the width and height of the target, reducing the interference information passed to the tracking algorithm. In addition, the regression-based training method can also reduce the frequency of redetection by 30% on average when blurring occurs. To further analyze the effectiveness of the loss function of the proposed regression, it is applied to another deep reinforcement learning-based tracker ACT, and the experimental results are shown in Figure 9. It can be found that the proposed method obtains an accuracy improvement of 1.2% and 1.3% on the OTB100 and OTB2013 datasets, respectively, and also improves the success rate. This is mainly due to the fact that the proposed method is more sensitive to target state changes.

For the target tracking motion module, a new deep reinforcement learning-based target tracking method AEVRNet with a nonconvex optimized random variance descent gradient and adaptive exploration strategy is proposed. First, the adaptive motion exploration strategy is proposed to combine time and space relations to extend the motion space to enhance the motion exploration capability and realize the tracking algorithm to jump out of the local optimal solution. Second, a nonconvex optimized stochastic variance descent gradient backward propagation method is proposed to optimize the supervised learning and deep reinforcement learning process of target tracking, which avoids the occurrence of premature convergence to the local optimal solution and thus obtains better convergence accuracy and speed. Third, an action reward loss function is designed for target tracking by regression, which is more sensitive to various aspects of the target state, such as the width and height of the target, and can further improve the accuracy of AEVRNet. In future work, attentive mechanisms with deep feature channels will be attempted to be introduced to improve the efficiency of feature utilization and enhance the robustness of the tracking algorithm.

## 5. Conclusion

With the popularization of the Internet lifestyle and the innovation of learning methods, more and more online learning systems have emerged, allowing users to study in the system anytime and anywhere. While providing convenience to users, online learning systems also bring troubles to users, who cannot quickly find the resources they are interested in from the huge amount of learning resources. In this paper, we apply deep learning to an English online learning platform and analyze learners and learning contents by clustering algorithm and association rules. Based on this, a content organization system is developed using genetic algorithms, which is applied to the case of this paper to provide learners with personalized learning content, with the hope that the system can be extended to other online learning platforms in the future. For the goal tracking algorithm update module, a deep reinforcement learning-based multitask multimodel goal tracking method is proposed, which is able to improve the tracking performance of the tracking algorithm on different tasks by using task-specific knowledge without increasing the parameters of the network model through knowledge migration techniques. For the adaptive student-teacher knowledge migration strategy, the teacher model can guide the student model to track online and migrate the task-specific knowledge to the online tracking student model through the network parameters. Specifically, the online student model is adaptively updated by the teacher model parameters and the online student model parameters together.

In the future, these models will improve the sense of efficiency and satisfaction of personalized recommendations of learning resources.

## Figures and Tables

**Table 1:** Table of model parameters.

*i*	*i*_1_	*i*_2_	*i*_3_
*θ*1	3	4	2
*θ*2	4	5	4
*θ*3	6	6	2
*θ*4	3	4	4
*θ*5	0	2	5
*θ*6	1	0	7
*θ*7	2	0	6

**Table 2:** Comparison of training and test sets for same target tracking.

Dataset	Category	Number of videos	Number of texts	Frame rate	Duration
OTB	5.06	20.11	15.32	18.03	14.37
VOT	5.32	19.48	15.24	18.14	14.60
TColor	5.03	20.16	14.33	18.04	15.66
Tencent	3.81	13.95	10.65	11.86	8.51
Lasot	4.98	18.73	14.26	16.20	12.14
MOL	59.72	61.88	60.36	61.30	56.44
GOT	6.26	19.78	16.09	18.63	14.46
VID_d	15.02	27.22	22.25	24.10	17.21

## Data Availability

The data used to support the findings of this study are available from the corresponding author upon request.
